# Multi-Compartment T2 Relaxometry Using a Spatially Constrained Multi-Gaussian Model

**DOI:** 10.1371/journal.pone.0098391

**Published:** 2014-06-04

**Authors:** Ashish Raj, Sneha Pandya, Xiaobo Shen, Eve LoCastro, Thanh D. Nguyen, Susan A. Gauthier

**Affiliations:** 1 Department of Radiology, Weill Cornell Medical College, New York, New York, United States of America; 2 Department of Neurology and Neuroscience, Weill Cornell Medical College, New York, New York, United States of America; Charité University Medicine Berlin, Germany

## Abstract

The brain’s myelin content can be mapped by T2-relaxometry, which resolves multiple differentially relaxing T2 pools from multi-echo MRI. Unfortunately, the conventional fitting procedure is a hard and numerically ill-posed problem. Consequently, the T2 distributions and myelin maps become very sensitive to noise and are frequently difficult to interpret diagnostically. Although regularization can improve stability, it is generally not adequate, particularly at relatively low signal to noise ratio (SNR) of around 100–200. The purpose of this study was to obtain a fitting algorithm which is able to overcome these difficulties and generate usable myelin maps from noisy acquisitions in a realistic scan time. To this end, we restrict the T2 distribution to only 3 distinct resolvable tissue compartments, modeled as Gaussians: myelin water, intra/extra-cellular water and a slow relaxing cerebrospinal fluid compartment. We also impose spatial smoothness expectation that volume fractions and T2 relaxation times of tissue compartments change smoothly within coherent brain regions. The method greatly improves robustness to noise, reduces spatial variations, improves definition of white matter fibers, and enhances detection of demyelinating lesions. Due to efficient design, the additional spatial aspect does not cause an increase in processing time. The proposed method was applied to fast spiral acquisitions on which conventional fitting gives uninterpretable results. While these fast acquisitions suffer from noise and inhomogeneity artifacts, our preliminary results indicate the potential of spatially constrained 3-pool T2 relaxometry.

## Introduction

Differential transverse relaxation rate (T2) of water in different brain tissues enables sensitive estimation of structural changes [1], [2]. Multi-echo T2 relaxometry is an MRI technique in which a small number of exponentially decaying components is fit to a series of T2-weighted images obtained at different echo times. The relative contributions of each tissue compartment is then inferred non-invasively, including the quantification of the water trapped between myelin bilayers, called the myelin water fraction (MWF), a quantitative map of myelin content in each brain voxel. MWF can be used to assess the integrity of white matter (WM) and it damage in neurological diseases like multiple sclerosis (MS) [3], [4], epilepsy [5], psychotic disorders [6], and Wallerian degeneration [7]. MWF has been shown to highly correlate with histological myelin measurement in animal models [8] and ex vivo brains [9]. Traditionally, clinical assessment of white matter disease is performed from T2-weighted sequences like Fluid Attenuated Inversion Recovery (FLAIR) images whose tissue contrast can frequently be quite high compared to currently available quantitative T2 methods. However, due to their non-quantitative nature and lack of anatomic or pathologic specificity, they do not meet the requirements of the next generation of quantitative brain imaging applications.

The prevalent fitting method, non-negative least squares (NNLS) [10], assumes a large number, up to 50 or 100, of components with known and regularly spaced T2 values, since the relaxation times of different components in unknown a priori. Far fewer observations (∼20–30 echo times) are available due to scan time considerations, which yields a highly under-determined and ill-posed problem whose solution suffers from non-uniqueness, noise amplification and instability [11]. Tikhonov regularization [12] of T2 relaxometry [10] added a stabilizing L2 norm penalty and helps ensure a unique solution, but is still noise-sensitive, frequently produces visually chaotic and diagnostically uninterpretable MWF maps. These challenges - stringent SNR requirements (leading to prohibitively long acquisitions or limited brain coverage), instability of results – greatly impeded the clinical utility of NNLS. Taking more echoes can improve the situation [11], [13] but is not a viable option due to excessive scan time [14].

The challenges in NNLS-based T2 relaxometry arise not only from the innate ill-posedness of the inverse problem [15], [16], but also from the introduction of an unnaturally large and biologically unrealistic number of relaxing components – a highly under-determined problem that frequently causes the NNLS algorithm to miss actual peaks in SNR-challenged data [17]. Theoretical considerations suggest that there is no scope for deducing more than 2 or 3 T2 components separated by a minimum interval from 15–20 echoes [18]; these criteria are violated by the NNLS algorithm. The choice then is between a highly underconstrained but linear estimation problem, and a constrained but highly non-linear problem, where only a small number of unknown compartments are sought to be fit to data.

In this paper, we eschew the underdetermined but linear NNLS approach in favor of a non-linear formulation that recognizes only 3 distinct T2 compartments in the brain, each exhibiting not a single T2 but a wide peak distributed within a small disjoint T2 range. This drastically reduces the number of unknowns, greatly improves the conditioning of the inverse problem and circumvents the biologically unrealistic and numerically indefensible need for 50 or 100 T2 compartments. A previous comparison between a similar 3-pool fit and NNLS demonstrated significant improvement in simulations [17]. Our approach is similar, but in addition, seeks to further improve conditioning by imposing a priori constraints. We therefore propose a *multi-voxel spatial regularization* approach which imposes two constraints:

1. There are only 3 distinct T2 pools in the brain – a fast relaxing myelin water pool, a slower intra/extra-cellular water pool, and a very long relaxing cerebrospinal fluid (CSF) pool. Based on numerous observations of T2 distributions, we model the first two pools by Gaussian distributions, whose parameters (mean location, height and variance) are unknown and to be determined. The long CSF pool is modeled as a single pool with a single unknown T2 and strength. See Table 1.
2. T2 characteristics of constituent water compartments change smoothly within coherent brain regions such as healthy WM while the boundary sharpness between distinctive regions should also be preserved. These spatial constraints are imposed separately on each parameter in the above multi-Gaussian model.

**Table 1:** List of parameters to be fitted, per voxel.

Symbol	Feature	Initial guess	Allowable range [lower, upper]
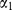	Height of myelin water distribution	0.1	[0, 1]
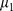	Mean of myelin water distribution	15 ms	[10 ms, 40 ms]
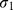	St. dev. of myelin water distribution	10 ms	None specified
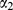	Height of intra/exra-cellular distribution	0.9	[0, 1]
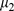	Mean of intra/exra-cellular distribution	80 ms	[60 ms, 200 ms]
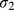	St. dev. of intra/exra-cellular distribution	100 ms	None specified
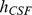	Height of long-T2 (CSF) signal	0	None specified
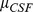	Mean of long-T2 (CSF) signal	1800 ms	None specified

Their initial guess and allowable range, used during constrained optimization, are also shown.

**Figure 1:**
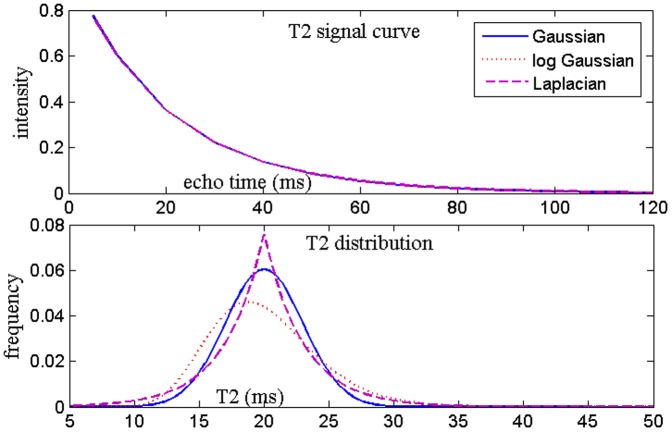
Numerical simulations and its effect on decay curve. Numerical simulation of various T2 distributions of roughly equal variance centered near 20– their effect on the resulting decay curve is minimal. Echo times are the same as those for in vivo MESE scans (omitting the highest points for ease of visualization).

Previously, a single Gaussian distribution was found to outperform monoexponential models in brain tissue [19] – here we have extended this to multiple Gaussians. The choice of Gaussian shape is motivated by simplicity and the fact that shape is relatively unimportant (see Figure 1, Discussion). For spatial constraints we adopt the approach first proposed in [14], by formulating an objective function which penalizes both the fitting error and non-smooth solutions. Unfortunately, their method is computationally challenging, taking up to 2 hours per slice, as it involves joint minimization over multiple voxels, each having about 50 unknowns. Our formulation has fewer unknowns but more challenging numerically due to non-linearity, which we solve using non-linear least squares [20] algorithm. We make several algorithmic innovations to improve the numerical efficiency. First, unlike [14] we eschew the explicit computation and storage of large matrices, instead performing iterative conjugate-gradient-based searches which are memory-light. We pre-compute the sparse Jacobian pattern of the non-linear least squares problem, which greatly speeds up computation. Since our regularization parameters are spatially invariant we only do the optimization once, unlike NNLS which requires a separate optimization for many regularization parameters in order to evaluate the chi-squared criterion. Due to these reasons our implementation is around 5 times faster than a local implementation of NNLS. However, we stress that the main objective of the new method is not a faster algorithm, but one that overcomes the limitations of ill-conditioning by utilizing spatial and model-based constraints.

We thoroughly exercise and numerically characterize the proposed algorithm using a variety of numerical lesion and whole brain simulations, MR phantoms and in vivo brain data acquired with the multi-echo spin echo (MESE) sequence. We demonstrate our method on a small number of normal subjects and a very large cohort of 154 MS patients, and demonstrate significant improvement over conventional fitting. We also present preliminary results on a new 3D multi-echo fast T2-prepared spiral imaging sequence [21], which provides noisy but very fast whole brain coverage and scan time of 10 min at 3T, making it a clinically feasible alternative to conventional MESE. While the conventional NNLS method gives noisier and less reproducible results on all datasets compared to our method, it is especially problematic on the fast spiral data, giving noisy, spatially incoherent and unstable MWF maps, whereas the proposed algorithm continues to produce clinically useful MWF maps. Thorough statistical analysis of whole brain variability, lesion detection, and region of interest analysis over 154 patients are presented.

## Theory

### T2 Relaxometry

Given MR signals y_k_ in a voxel measured at echo times TE_k_ (k = 1,…, K), a set i = 1,…,N of discrete sub-components are hypothesized to exist, each producing an exponentially decaying signal α_i_exp(-TE_k_/T2(i)) with a known T2 constant of T_2_(i) and an unknown volume fraction of α_i_. Assuming a slow exchange regime [22], this gives:
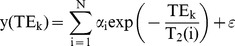
(1)


or equivalently, **y**  =  **Ax** + **ε**, with 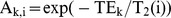. Vector **y** is a collection of data *y_k_* acquired at echo time *TE_k_*, **x** is a vector of the unknown *α_i_*, and **ε** denotes instrumentation noise. This system of linear equations is typically under-determined (N>K) and ill-posed, making a direct inversion either impossible or prone to extreme noise sensitivity, instability and non-uniqueness [23]. Tikhonov regularization [12] was proposed to partially overcome this problem by minimizing the cost function [24]

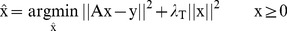
(2)


for each voxel using the non-negative least squares (NNLS) algorithm. Here *λ_T_* is a regularization parameter. We follow the approach and notation of a recent spatially constrained multi-voxel Bayesian minimization [14]: 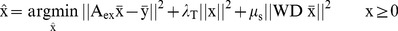
(3)


where single-voxel quantities **x**, **y** are collected in multi-voxel vectors, and the expanded (block diagonal) matrix **A**_ex_ is defined similarly. The last term penalizes spatially non-smooth solutions via the first-difference operator and corresponds to the convolution of the T2 distribution image with a high-pass filter. **W** is a diagonal weighting matrix which allows for variable penalties associated with T2 points.

### Proposed Multi-Gaussian Spatial Regularization

In the *j*-th voxel we wish to determine the following set of unknown parameters (Table 1):
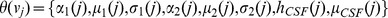



which in turn uniquely determine the T2 distribution as a sum of two Gaussians and a long-T2 signal:
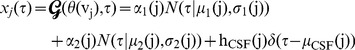
(4)


Each Gaussian is denoted by the shorthand *N*(.), and T2 distribution is over variable τ. The T2 distribution above resulting from the multi-Gaussian model is evaluated on the 40-point range of T2 values, and the delta function above turns into a discrete delta. Collecting multi-voxel parameters in the vector **θ**  =  {*θ (v_j_), j* = 1,…, *N_v_*}, and relating the Gaussian parameters to the resulting T2 distribution by x  =  G(θ), we minimize 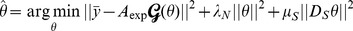
(5)


The 3rd term enforces spatial consistency constraint #2 above, separately on each parameter of the model, via the first difference operator matrix *D_S_*. The global regularization parameters *λ_N_* and *μ_S_* are unknown *a priori* and their optimal values are chosen by a semi-supervised procedure (see Method). Once minimization has concluded, we obtain the final T2 distribution
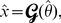
(6)


Since we model pools explicitly, MWF calculation does not involve integration: 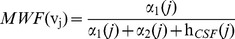
(7)


under the constraint that should be in the range during the minimization of Eq (6). The operator 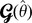 is non-linear, hence Eq (5) is non-convex. We formulate it as a non-linear least squares problem by combining all 3 terms into the L2 norm of a single vector given by
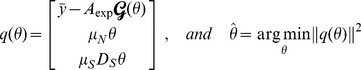
(8)


Eq (8) is merely a reframing of Eq (5), and is intended to stress that our chosen approach relies on solving the non-linear least squares problem (8) rather than the general problem (5), and takes advantage of the 2-norm structure available to us. This approach can take advantage of the fact that at each iteration, the minimization problem is a least squares problem around a local Jacobian 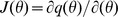
which is quickly solved by preconditioned conjugate gradients algorithm.

## Methods

### Simulating healthy brain MWF

A synthetic MRI brain image and its white, gray and CSF probabilistic masks obtained from the Montreal Neurological Institute (MNI) were used to simulate multi-echo T2 data, each tissue class having a unique T2 signature. The myelin fraction was set at 14.5% for pure white matter, 4.5% for pure gray matter, and 0% for CSF. The myelin, intra/extra and long-T2 pools were simulated as Gaussians centered at 25 ms and 100 ms, respectively, and the CSF peak was set at 1800 ms. These pure T2 components were weighted by the probabilistic masks to get the actual components. Additive white Gaussian noise was introduced at SNR level varying from 100 to 1000; SNR is defined for this purpose with respect to the signal at first echo.

### Doped water phantom experiments

10 test tubes filled with water doped with varying concentrations of Gadolinium and MnCl2 were scanned using the MESE sequence at 1.5T over 8 slices and processed using the propsoed algorithm. The nominal T2 relaxation times were measured by fitting a single exopnential onto the averaged signal curves from all voxels in each test tube.

### Human Imaging

Human study with full informed consent was approved by Weill Cornell Medical College Institutional Review Board. Consent was obtained both in oral and written form with detailed documentation. Spin echo T2 data were obtained from 2 healthy volunteers at 1.5T, and a recent whole brain 3D T2prep spiral scans [21] at both 1.5T and 3T GE HDxt scanner. The imaging parameters were: Spin echo: axial FOV = 30 cm, matrix size = 256×128 (interpolated to 256×256), partial phase FOV factor  = 0.6, slice thickness  = 5 mm, number of slices  = 12, TR = 2500 ms, echo spacing  = 5 ms, number of TEs  = 32 (5, 10–310 ms (10 ms step)), scan time  = 38 min. 3D T2prep spiral: axial FOV  = 24–30 cm, matrix size  = 192×192 (interpolated to 256×256), slice thickness  = 5 mm, number of slices  = 32, TR = 2500 ms, flip angle = 10°, number of TEs = 15–26, scan time  = 10–26 min. Two end slices were discarded to account for imperfect slab excitation profile. Skull (very short T2) and fluid (very long T2) signals were removed using BrainSuite v09.

Next, a large study of **154 MS patients** was undertaken at 3T under the 3D T2prep spiral sequence. This cohort was used in this paper for the purpose of obtaining whole brain statistics and assessment of variability – a detailed study of pathological discrimination on MS will be carried out in future work. To this end, a regional parcellation was imposed on T2 images using the labeled Freesurfer atlas [25] using a recently developed pipeline in our laboratory [26]. Regional averages of MWF for gray matter and adjoining white matter regions were computed and their histograms created. A few of these patients were chosen, on the basis of visually apparent focal WM hyperintensities, for a preliminary neurological assessment of demyelinating WM lesions for the purpose of illustrating the lesion discrimination ability of the algorithm. T2-weighted volumes and where available, FLAIR volumes were co-registered to their MWF maps. Hyperintense focal lesions were manually segmented on the co-registered volumes under a neurologist’s (SAG) supervision. These lesion masks were mapped onto MWF maps, and voxel-level statistics were computed.

### Implementation of Multi-Gaussian Spatially Constrained T2 Relaxometry

The list of 8 parameters are shown in Table 1, along with initial guess and allowable range, which were chosen based on prior experience. Forty T2 points logarithmically spaced over a range of 5–300 ms were chosen for the T2 distribution. Multi-voxel data fitting was implemented using the iterative **lsqnonlin** algorithm [20] in MATLAB version R2011b running on a Windows 7 64-bit platform and Intel core 2 Duo processor with 2.2 GHz, 4 GB memory. All voxels in the 3D volume were solved for at once, presenting a very large minimization problem necessitating considerable effort over efficient algorithm design. Since the Jacobian J(θ) is not expressible in closed form, it needs to be estimated at each iteration from first differences over 8·N_V_ variables – a daunting memory requirement. Fortunately, J(θ) is highly sparse due to the structure of q(θ), and its sparsity pattern was pre-computed and passed to **lsqnonlin**. Memory requirements for storing voxel neighborhood system and Jacobian was minimized by using sparse data types and a rewrite of the vendor-supplied **lsqnonlin**-related functions involving pre-allocating some large arrays. The final code was able to achieve 10–20 times speed improvement over a direct call to **lsqnonlin**.

### Optimization of regularization parameters

Unfortunately choice of regularization parameter greatly affects the outcome, hence our NNLS implementation evaluated the chi-square criterion [24] at 100 *λ_T_s* spaced logarithmically within [10^−1^, … , 10^−5^] for each voxel, and one that gave residual norm between 102.0%–102.5% of the non-regularized residual norm was selected, as specified in previous NNLS reports [13]. Unfortunately, this problem is even more acute for the proposed approach due to strong non-linearity, hence we developed a novel algorithm design which greatly reduces parameter dependence by incorporating the chi-squared criterion within the minimization itself. At each iteration we rescale the prior penalties such that the ratios 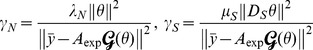



are held constant throughout the minimization, as follows. We start with 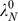 and 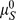 and at each iteration *n* we introduce the following rescaling:
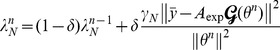





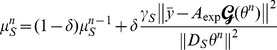



The 2^nd^ term is the fully rescaled version which maintains constant, but in order to reduce the potential for instability or oscillations, we introduce a small step size to prevent large discontinuous jumps. This approach completely obviates the need for testing large ranges of regularization. We tested all cases with the same prior ratios γ_N_ = 0.1, γ_S_ = 0.1 and this gave consistent results across subjects, however in some cases slight tweaks to these presets produced somewhat better results.

### Regional Statistical Analysis

Masks of WM and GM tissue classes were obtained using SPM8 software [27] by co-registering the 10 MS subjects’ T2 images to T1, then to the T1 template from the Montreal Neurological Institute (MNI). These transformations are applied to the computed MWF maps. Spatially unbiased whole brain regional analysis was performed by parcellating the cortex into 68 regions using pre-labeled MNI atlas. Mean MWF values are computed for each region, split further into the cortical ribbon and adjacent white matter. From these mean ROI values, intra-class coefficient of variance (COV) was computed and between all region pairs a two-tailed paired-sample t-test was used to assess p-values, with p<0.05 considered statistically significant.

## Results

### Brain Simulation Result

**Figure 2:**
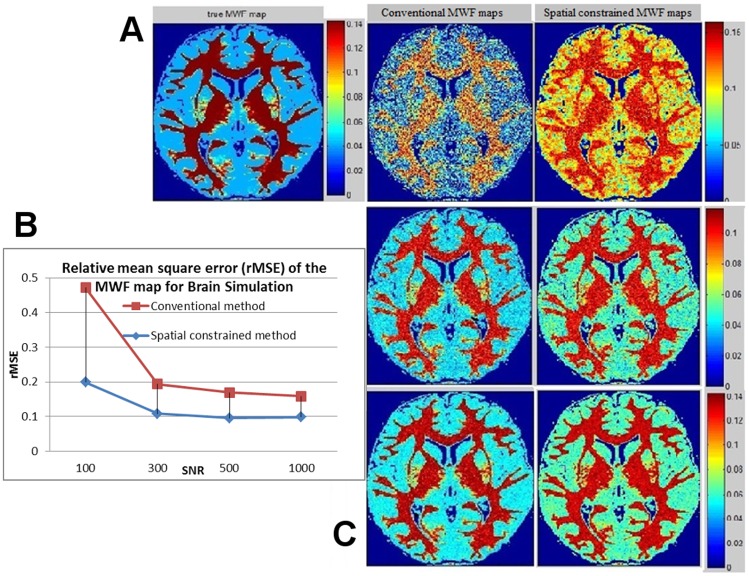
MWF maps generated by simulation of MESE data using brain phantom. Brain phantom (A) was used to simulate MESE data, and rMSE was calculated at different SNRs (B). The corresponding MWF maps are shown in C at SNR = 100, 500, 1000 (top to bottom).

Our purpose was to quantify error rate and to explore the blurring effect of spatial regularization at tissue boundaries. MWF maps in Figure 2 indicate higher accuracy and less noise using our technique. The relative mean square errors 
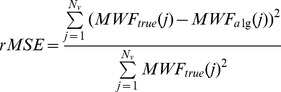
 of MWFs is also shown, where *N_V_* is the total number of voxels, and *MWF_true_* and *MWF_alg_* are the true and estimated MWF. There is little evidence of edge blurring, in fact we see higher tissue contrast than conventional map. Figure S1 and S2 in File S1 characterizes the effect of spatial regularization parameter *μ_S_* at various SNR levels, exhibiting the typical U-shaped behavior: low *μ_S_* gives too much noise, high *μ_S_* too much smoothing; an intermediate value provides the best numerical and visual compromise – here this is achieved at *μ_S_ = 0.01*. The corresponding MWF maps visually reinforce this impression. Contrary to expectation, the highest *μ_S_* did not produce the smoothest image, probably due to the realization of a different local minimum of the non-convex cost. Note that this simulation was not designed to investigate intra-tissue spatial variations; although it is possible to construct numerical phantoms for this purpose, we feel this is best investigated via *in vivo* data. Further, the use of 3 pools in the simulation might appear to inherently favor the multi-Gaussian model, but the comparison with conventional NNLS, which is agnostic to the number of pools, is still valid – the experiment was designed precisely to test the additional benefit of applying a realistic multi-pool model.

### MESE doped water phantom results at 1.5T

**Figure 3:**
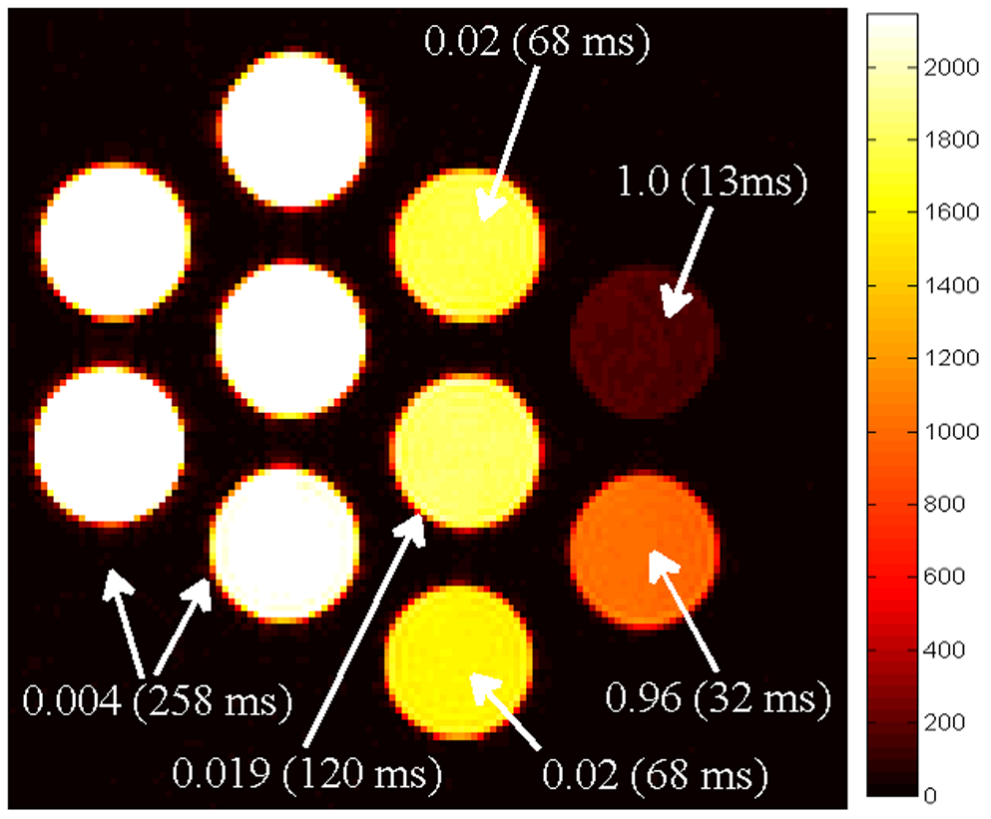
MESE experiment using test tubes. MESE experiment at 1.5T on 10 test tubes filled with water doped with varying concentrations of Gd and MnCl2, hence varying T2 times measured by single exopnential fit. Raw MR signal intensities of a middle slice of the 5^th^ echo (40 ms) are shown using idicated color scale (arbitrary scanner units). The text arrows show the average MWF estimated by the propsoed algorithm within each test tube and its nominal T2 in parentheses. The “true” MWF would be 100% for the two right-most tubes, and 0% for the rest. These results indicated good accuracy of our agorithm on MESE data.

Figure 3 shows the 5^th^ echo image (40 ms), the average within each test tube of our MWF, and their measured T2 in parentheses. Note that this doped water phantom does not produce multiple pools – only differentially shortened single T2 pool. Thus, the purpose of this experiment is to assess accuracy and bias when the multi-pool assumption is violated. Clearly, our algorithm achieved quite good accuracy on this data, yet a small bias exists which is more prominent for T2 values close to the 40 ms myelin cutoff.

### *In vivo* MRI Results

Numerical convergence shows a classic pattern (Figure S3 in File S1). Only 20 iterations appear sufficient, and further iterations do not appreciably reduce the cost or image appearance. We chose 50 iterations for all cases. The processing times of 9 slices of size 192×192 pixels were approximately 4 hours with the conventional method and 25 minutes with the spatial constrained method. For 8 slices of size 256×256 pixels, the processing times were approximately 6 hours with conventional and 1.2 hours with spatial algorithm.

### Healthy MESE data

**Figure 4:**
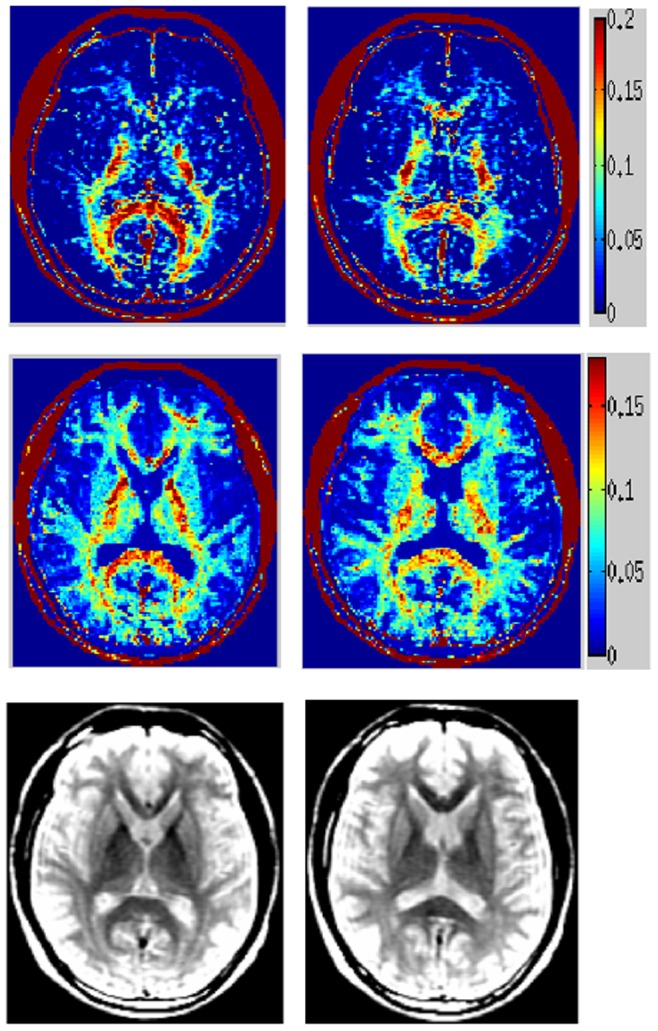
MWF maps of healthy MESE scans acquired at 1.5T. MWF computed from healthy *in vivo* anisotropic MESE scans acquired at 1.5T. Conventional reconstruction (top), spatial constrained reconstruction (middle) and anatomical T2 FLAIR images (bottom). Two consecutive axial slices at the level of thalamus and putamen are shown.

Figure 4 shows proposed MWF maps from a healthy subject’s MESE scan, demonstrating a clear reduction in noise, improved spatial coherence and depiction of WM structures compared to NNLS. The proposed map encompasses a much larger area of WM, including frontal and lateral projections fibers which were silent on the conventional map. As an anatomic reference, the bottom row shows T2-prep spiral images at 50 ms. Table 2 summarizes MWF measured on the whole brain and at selected WM ROIs of normal subjects. At least 20 voxels were used in each ROI for computing these statistics. The last two rows show ROI comparison with published literature: voxel-based analysis (VBA) [28] and multi-voxel Bayesian (MVB) [14]. Although a gold standard is not available, our values are within the published range and seem to roughly match NNLS.

**Table 2:** MWF of selected WM ROI obtained with proposed and conventional methods, from both MESE and spiral sequences.

	Genu of Corpus Callosum	Splenium of Corpus Callosum	Internal capsules
Conventional: MESE 1.5T	18.1±2.5	18.3±1.6	8.89±4.9
MultiGaussian: MESE 1.5T	18.5±4.0	16.8±2.1	10.8±4.0
Conventional: T2prep spiral 3T	24.4±5.1	18.4±1.6	18.1±3.0
MultiGaussian: T2prep spiral 3T	19.7±0.9	17.6±0.8	18.3±1.5
**VBA**	**10.2±0.2**	**14.4±0.2**	**17.2±0.2**
**MVB: FSE 1.5T**	**16.7±1.8**	**14.6±3.1**	**14.2±1.5**

The last two rows show comparison with published literature: VBA [28] and MVB [14].

### Healthy T2prep spiral data

**Figure 5:**
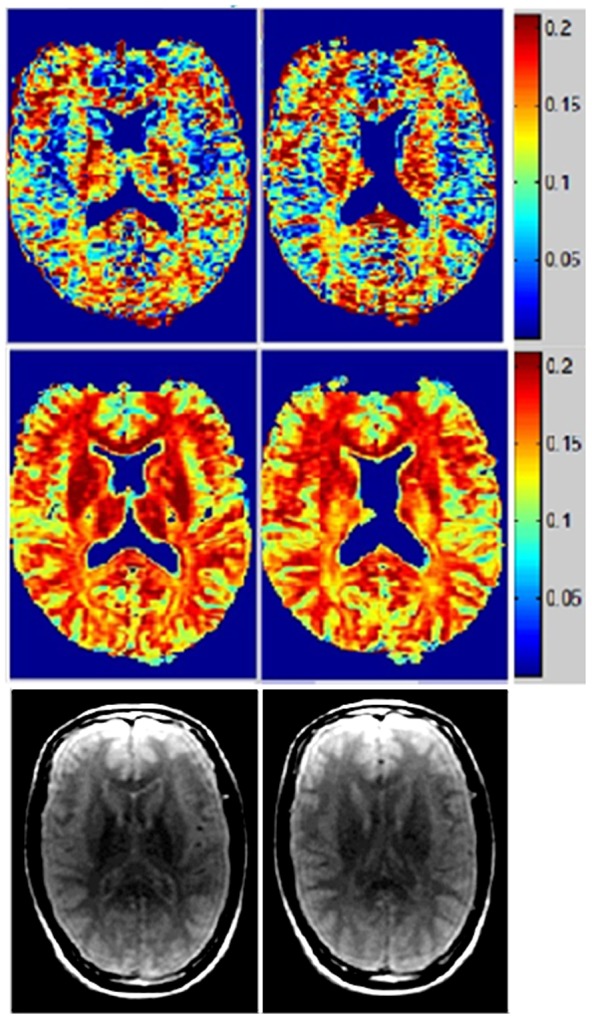
MWF maps of healthy 3D T2prep adiabatic spiral brain scan acquired at 1.5T. Two axial slices of MWF computed from the second healthy *in vivo* 3D T2prep adiabatic spiral brain scan acquired at 1.5T, with conventional reconstruction (top), spatial constrained reconstruction (middle) and anatomical T2 weighted images (bottom).

**Figure 6:**
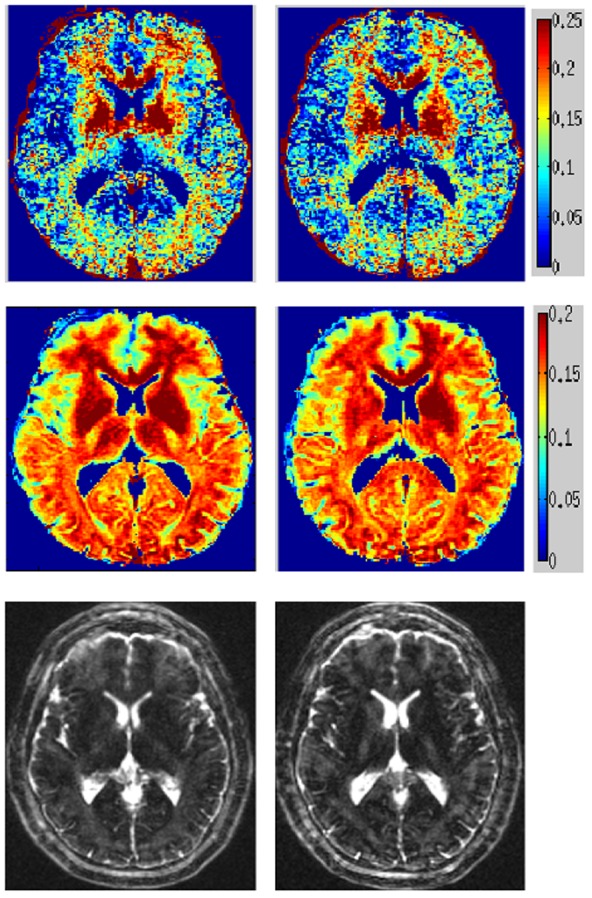
MWF maps of another healthy 3D T2prep adiabatic spiral brain scan. Another healthy brain 3D T2prep adiabatic spiral example, with MWF (top) and T2-weighted image (bottom) of two adjacent axial slices.

**Figure 7:**
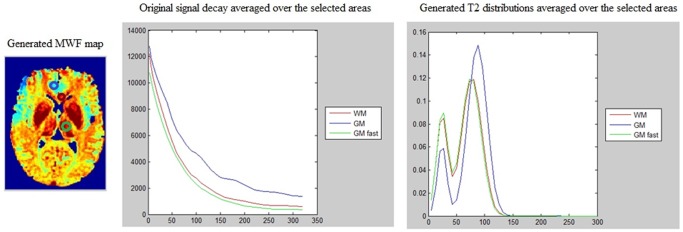
Examination of elevated MWF, relaxation curves and T2 distribution. Detailed investigation of elevated MWF in deep gray matter. Relaxation curves for three brain areas are shown on the middle panel: WM, GM and deep brain GM – these areas are denoted by circles on the MWF map generated from spatial constrained method (left panel). Dark red circle indicates the WM area, blue indicates the GM area, and green indicates the GM with fast relaxation rate area. The fitted T2 distribution from each region is shown in the right panel - curves correspond to the ROI average

Figure 5 shows processed MWF maps from T2prep spiral scans of a healthy volunteer, which has a different appearance compared to MESE, due to spatial inhomogeneity and other artifacts introduced by the fast sequence. Although this has made the NNLS method practically unusable, our approach is able to retrieve anatomically faithful myelin maps from this data. However, there is upward bias in GM, which should be lower than depicted (see Discussion). The values in WM, however, are in line with reported numbers, as indicated in Table 2. Figure 6 shows another example with similar characteristics, but it additionally illustrates an oversized effect of T2 shortening induced by iron deposition in the basal ganglia (see Discussion). The difference in MWF between whole brain WM and GM was significant in all subjects (p<0.001) for both methods, indicating good tissue differentiation. Corresponding COV data (Table 3) shows greatly reduced variability from our algorithm, reinforcing the earlier visual impression of smooth, noise-free maps. Figure 7 shows typical T2 decay curves from WM, GM and iron-rich deep GM and their estimated T2 distributions – they show expected behavior, with fast initial decay in WM (and iron rich nuclei).

**Table 3:** Comparisons of Mean Coefficient of Variance (COV) of MWF obtained for the T2prep spiral sequence in various ROIs between proposed and conventional methods.

Region	Conventional COV	Spatial COV
Whole brain WM	0.84	0.14
Whole brain GM	1.14	0.24
Genu of corpus callosum	0.21	0.05
Splenium of corpus callosum	0.09	0.05
Internal capsule	0.17	0.08

### T2prep spiral data of MS patients

**Figure 8:**
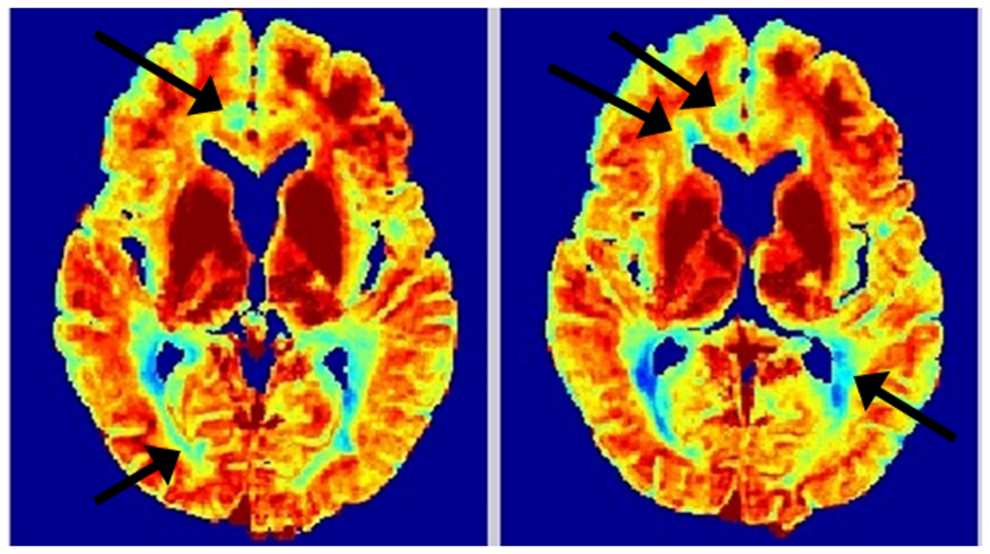
Illustration of demyelinating lesion in MWF map of a MS patient. Two axial slices of proposed MWF map of a MS patient. Note the excellent depiction of demyelinating lesions (arrows) and improved definition of callosal and peripheral white matter.

**Figure 9:**
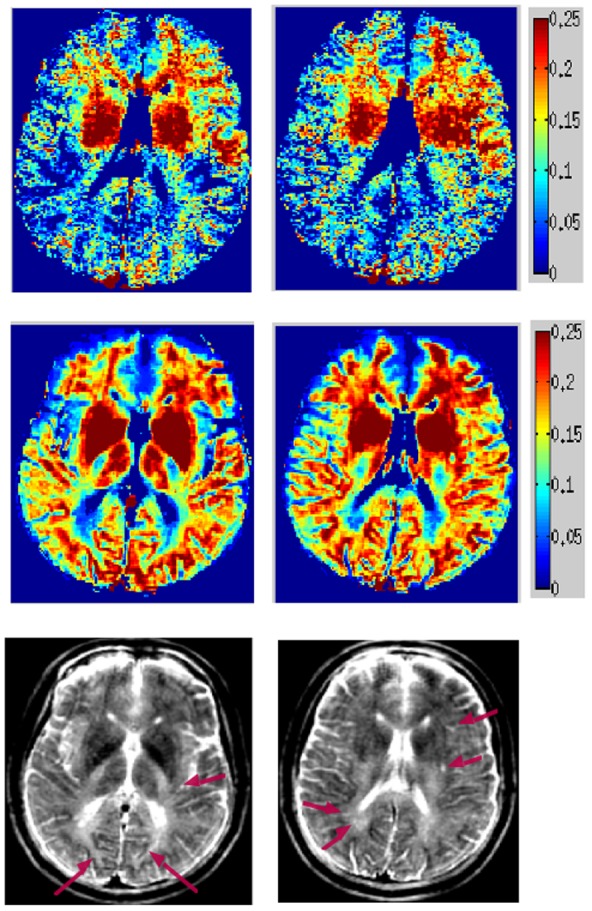
MWF maps of MS patient using conventional and spatially constrained method. MWF maps computed from *in vivo* MS patient scan. Top to bottom are MWF maps from conventional method, spatial constrained method and T2-weighted image at 60 ms. Arrows in T2 of point to small, focal lesions, externally confirmed on FLAIR. These lesions are well delineated on the spatial map but not on conventional map.

The example in Figure 8 displays exquisite depiction of periventricular WM injury (arrows). The NNLS map was uninterpretable like Figure 4 and is not shown. There was however, a prominent anterior-posterior spatial gradient. Figure 9 shows an MS example with both small, focal lesions and diffuse ones, along with T2 weighted images at 60 ms which are hyperintense in lesions. Another example of MS lesions is in Figure S4 in File S1, with external validation of lesion location and extent derived from coregistered FLAIR data. Visually, our method differentiated lesions more clearly than the noisy NNLS maps. Spatial smoothing did not obscure small lesions. Table 4 shows p-values from student’s t-test between normal WM and lesions (drawn manually from FLAIR under a neurologist’s supervision) of MWF from 3 MS patients – our approach has higher detectability of demyelinating lesions.

**Table 4:** P-values of MWF difference between normal WM and WM lesion detected by two methods.

	P-value	
	Conventional	Spatial
Patient 1	0.15	0.023
Patient 2	0.041	<0.001
Patient 3	0.067	0.042

### Whole brain and regional MWF Statistics

**Figure 10:**
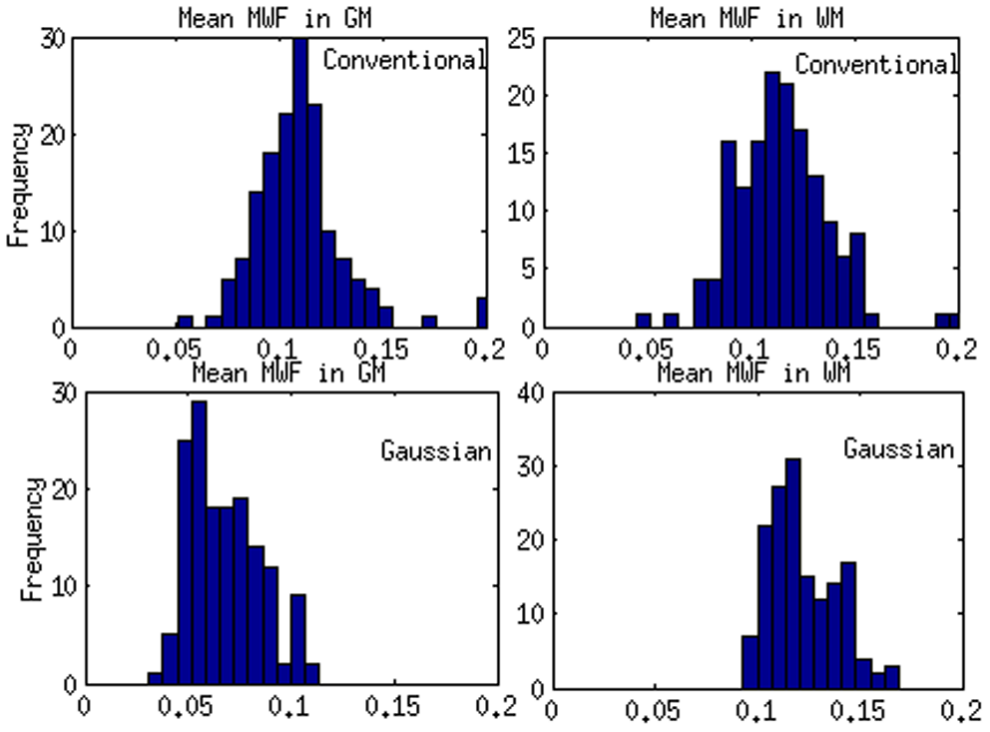
Histogram of MWF for all the subjects and t-test between WM and GM. Histogram from different subjects of MWF averaged over all regions. A t-test between the WM and GM groups yielded p<10^−6^ for multi-Gaussian and p = 0.0121 for conventional algorithm.

**Figure 11:**
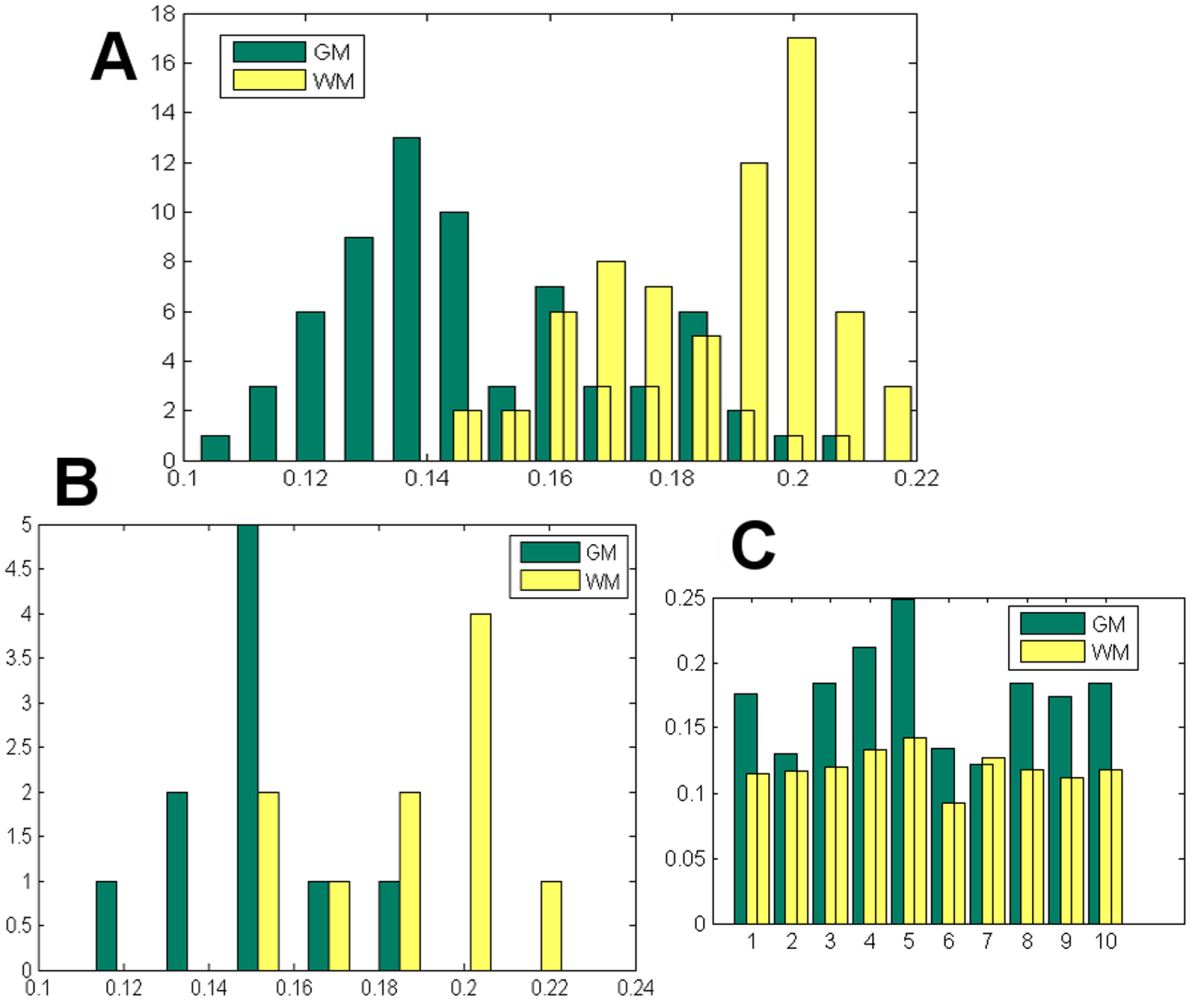
Histogram and COV of regional MWF, histogram averaged over all regions. (A) Histogram of regional MWF of each ROI averaged over 10 MS subjects. MWF in WM has a higher distribution than GM, and the two are statistically well separated, with p<0.001 from a student’s t-test between the WM and GM regions (B) Histogram from different subjects of MWF averaged over all regions. A t-test between the WM and GM groups yielded p<0.001. (C) COV of regional MWF for each subject, indicating low variability in regional variations in both WM and GM.

Whole brain statistics across all 154 MS patients is shown in Figure 10. Histograms of the distribution of white matter and gray matter MWF are shown for both the proposed and conventional NNLS methods. It is clear that there is high statistical separation between white and gray in our results, much more so compared to the conventional method. Statistical significance (p-values) is noted in caption. Regional averages of MWF of the smaller sample of 10 MS patients for 84 gray matter regions from the Freesurfer atlas and adjoining white matter regions are presented in Figure 11: histogram of regional MWF averaged across all subjects (A), histogram of whole brain GM and WM over all subjects (B), and COV between different regions for all 154 MS patients. The latter was quite small (0.1–0.2), which indicates consistent and spatially homogeneous estimates of MWF. Note that although both Figures 10 and 11 appear to show MWF statistics of GM and WM regions, the former is evaluated on all voxels, while the latter on regions. In either case the histograms show good separation between white and gray matter, compared to the conventional NNLS method. Despite upward bias in GM MWF from our algorithm, the statistical separation of the MWF distribution in WM and GM is very good, with p<0.01.

## Discussion

### Summary and impact of main results

This study demonstrates that physically realistic multi-Gaussian modeling and spatial constraints can overcome the limitations of conventional NNLS. The algorithm is fast due to efficient iterative, low-memory design. The method was thoroughly exercised and numerically characterized using a variety of experiments: numerical lesion and whole brain simulations to assess spatial coherence and edge preservation; doped water phantom to assess accuracy and bias in single pools where the model assumptions are violated; two different in vivo sequences - spin echo and T2-prep spiral; two scanner field strengths – 1.5T and 3T; and both healthy and disease. In particular, our disease cohort was very large, consisting of 154 MS patients. Although any given experiment only tells a partial story, the combined impact of all these experiments presents a robust picture of our algorithm compared with currently available alternatives. Simulated and MRI data show visually and numerically improved MWF measurements, less noise, greater spatial consistency, faithful resolution of small WM feature, higher WM/GM differentiation, and higher lesion detectability. The variability in MWF (COV) is substantially reduced. White matter coverage is significantly larger, including frontal and lateral projection fibers which are frequently silent in NNLS. In MS patients the visual depiction and statistical discrimination of lesions is substantially enhanced.

Our work provides a clear path to practical utility in applying quantitative relaxometry in the clinic, as compared to traditional visual assessment on FLAIR images, which are not quantitative. Our algorithm has the potential to produce quality whole brain myelin maps in under 10 minutes when applied to fast but noisy T2 prep spiral sequences on which NNLS fails. The spiral results meet the clinical goal of whole brain MWF maps in under 10 minutes scan time, and can be invaluable in diagnosis, prognosis and longitudinal monitoring of efficacy of treatment-induced remyelination. Fast processing time could enhance clinical and routine applicability – however this was not the focus of current work. Implementation on high-end multi-core server hardware could potentially reduce processing time to just a few minutes – which would open up several interesting clinical applications which are currently impeded due to overly long execution time of existing algorithms. While simulation, phantom and in vivo results on conventional MESE scans indicate little bias, our MWF maps from fast spiral scans show upward bias in GM and spatial inhomogeneity, which merit further effort in sequence design. A modest level of residual bias, where a non-zero myelin fraction is reported when there is none, is expected behavior for model- and constraint-based methods when the model assumptions are violated severely.

### Model Justification

#### Three pools

What if there are more than 3 compartments, for instance a slowly relaxing component between 300 ms and 800 ms [29]. Our model already allows for a long-T2 component, which could, if required by the data, conform to this intermediate compartment. Our algorithm design could easily accommodate a fourth compartment, but we think it inadvisable. Due to scan time limitations, sampling echo times higher than 300 ms is practically difficult. This limits the number of compartments above 300 ms that can be reliably discerned. Even if more echoes could be acquired, numerical conditioning of the inverse problem imposes stringent limits on the maximum number of compartments that can be reliably estimated [18]. In terms of biophysics, we find no strong justification or clinical interpretation of four compartments.

#### Gaussian peak shape

Although our method can easily accommodate any parsimoniously parameterized peak shape, the choice of Gaussian was motivated by simplicity, ubiquity and prior reports [19]; specifically we eschewed prior use of log-Gaussians [2] for computational reasons. What if the data preferred other shapes, or worse, require arbitrary model-free shapes? Certainly, one of the main attractions of full spectrum T2 relaxometry is its ability to deduce arbitrary, non-discrete T2 distributions [13]. A relatively under-appreciated factor is poor numerical conditioning, which leads to a very large number of possible T2 distributions all fit the multi-echo data equally well, hence small differences in the shape profile will be simply invisible to the data. This is illustrated in Figure 1, which shows 3 differently shaped but roughly equally dispersed distributions centered near 20 ms and their decay curves: Gaussian, log-Gaussian and Laplacian. The decay curves for these disparate peaks are strikingly similar and the discrepancy between them vanishingly small. Given typical levels of MR noise and artifacts, there is no hope for successfully estimating the precise shape of the true T2 distribution beyond low-order statistics like mean and variance, for which Gaussians are the canonical distribution. Worse, regularization can itself greatly influence the estimated distribution, making anything beyond the second moment operationally suspect.

#### Spatial constraints

These may potentially blur edges and small lesions or other pathology, but this can be prevented by a conservative choice of regularization parameter. On the contrary, spatial constraints can *improve* the definition of small lesions by preventing noise from occluding the lesion, as shown later by our results on demyelinating diseases. **Additive noise model**. In this work we have assumed Gaussian white additive noise, but a strong case can be made for Rician noise, especially in late echoes which have generally low signal levels. However, as is well-known, such models result in more challenging objective functions, necessitating more advanced minimization strategies which may not be amenable to least squares formulation used here. Given that spatial constraints already increase the numerical complexity of our approach manifold, we chose to stay with the Gaussian assumption in the interest of computational cost. This is in keeping with the vast majority of prior work in this area, where Gaussian assumption is routinely and successfully employed even in cases where it might be sub-optimal.

### Comparison with related approaches

Models involving 2 or 3 pure relaxing T2 components already exist [17], but here we accommodated more realistic dispersed Gaussian distributions to capture micro-environmental heterogeneity within tissue compartments. The information contained in peak dispersion is lost in pure multicompartment models, including those fitted to steady-state acquisitions like mcDESPOT [30], [31]. When applied to fast T2prep spiral data, our algorithm can provide a compelling alternative to mcDESPOT in terms of scan time but in addition handles dispersed peaks. Other alternatives for myelin quantification, like T2^*^ [32] or magnetization transfer [33] provide complimentary information regarding multiple tissue compartments, but due to differing mechanisms and pathological specificity they should be considered substitutes for multi-compartment T2 modeling.

Unlike the whole brain coverage we demonstrate, previous T2 relaxometry studies of MS are generally reported on selected slices [33], [34], hence preclude the quantification of diffuse or systemic pathologies. Our processing time is roughly 5 times shorter than local implementation of NNLS, increasing its routine clinical applicability. Significantly shorter processing time has been reported for NNLS [15] using more powerful hardware and parallel processing techniques, but the above comparison remains valid because presumably our method too could benefit similarly from hardware and parallelization. Our MWF maps have similar range as prior reports [10], [14], [16], but better visual quality in terms of smoothness and delineation of brain structures. The mean MWF values of major WM structures (Table 2) are consistent with the literature [3], [10], [15], [28], but our numbers are generally higher, e.g. in the genu of corpus callosum and in deep gray matter. There is also discrepancy in the internal capsule specific to MESE results, where both conventional and multi-Gaussian MWF values are low compared to all other data. We do not fully understand the reason, but note that the occlusion with basal ganglia makes ROI drawing difficult and operator dependent. There is no unanimity in the true value of MWF in normal brain tissue, hence it is difficult to assess accuracy on in vivo human data. In any case, absolute agreement in MWF numbers between studies is probably unrealistic due to differences in hardware, study subjects, pulse sequence, etc. Exchange between compartments might affect MWF. Interestingly, our results are similar to mcDESPOT [30], [31], both numerically and visually; in fact our numbers are generally intermediate between NNLS and mcDESPOT. Since both mcDESPOT and our multi-Gaussian approaches rely on pre-defining the number of distinct pools, this might perhaps explain the similarity in their visual appearance, and their distinct appearance compared to NNLS methods which are non-parametric. Until a clear in vivo gold standard emerges for human tissue, we must rely on statistical separation (rather than absolute MWF values) between WM, lesions and GM as the most practical way to assess clinical utility, as has been observed in various mcDESPOT studies. Our results are statistically very sensitive in detecting demyelinating lesions (Table 4) – much more so than NNLS – irrespective of the actual MWF values each method reports. Gray/white contrast is also well-preserved (Figure 10). Since COV values in Table 3 were computed over voxels, they are higher than some prior reports which used ROI averages [35].

Spatial constraints are prevalent in image processing [36]–[38] and MRI reconstruction [39], [40], but rare in T2 relaxometry. Spatially smoothed versions of conventionally obtained T2/T2* distributions were proposed as “reference distributions” and deviations from this was penalized [41] [42]. These approaches do not amount to a truly spatially constrained approach, because the inverse problem is still solved voxel-wise. They are unlikely to fundamentally solve the problem of ill-posedness, because the noise comes from the fitting process itself and must therefore be resolved *during* the fitting process by incorporating spatial constraints directly into the solution -e.g. see [39], [40]. Another potential problem is that the “reference distribution” obtained by NNLS can itself be inaccurate or uninformative (as shown in some of our examples), hence any estimate relying on this reference is prone to inappropriate bias and obliteration of genuine spatial variations. Our implementation of this approach failed on our spiral data and is not shown here. Since the T2 distribution of each voxel is given by a small number of tissue classes, it can be assumed to be sparse in the T2 range of interest, i.e. 5 ms to 300 ms. Therefore there is potential for using sparse reconstruction algorithms on this problem. Although this was not the focus of the current work, we were able to perform preliminary implementation and testing of sparse algorithms on this problem, by imposing and solving for L1-norm regularization terms. The details and results are contained in Supplementary Information S5 in File S1. The results contain therein are unimpressive, and suggest that a direct application of sparse reconstruction methods is likely to be unsuccessful. Our reasoning is that since sparsity is applicable on a single voxel basis, the sparse algorithm must be run one voxel at a time, hence by itself sparsity may not be not sufficient to overcome the ill-posedness of the problem. This is why our proposed approach favors both a model-based restriction to 3 classes, as well as additional spatial constraints. In contrast, our approach imposes weaker but realistic spatial coherence constraints.

The spatially constrained optimization proposed here is similar to our prior work [14], but the additional novelty here is regarding a constrained model along T_2_. Thus our method has far fewer degrees of freedom, where we have traded an unconstrained but quadratic cost function with guaranteed global minimum for a highly constrained but non-convex cost whose minimization is challenging. The risk of entrapment within inappropriate local minima and undesirable sensitivity to initial guess exist, but are outweighed by considerable gains, like unique solutions, better conditioning, and noise resistance. Our processing time and memory usage are a fraction of [14]. The MR sequence used herein is also quite different from that used to evaluate [14]. Given the difference in methodology and data acquisition, a comparison with [14] is unlikely to be relevant or useful, hence it is not shown in this paper. Instead we have directed our comparisons against the current benchmark method, NNLS, which is both widely used and well characterized.

### Limitations and future work

Our 3T spiral results suffer from apparent bias in GM, where we report higher numbers than previously reported. We attribute this to the spiral sequence rather than algorithmic issues, since the bias is not appreciable in simulations (Figure 2), phantom (Figure 3) or in vivo (Figure 4) MESE data. All MR acquisitions employed equal spacing between refocusing pulses to minimize the buildup of unwanted stimulated echoes [43] by maintaining a consistent refocusing efficiency across the excited 3D volume. Yet, T2-prep spiral acquisitions exhibit significant spatial gradient caused by B0 inhomogeneity, B1 bias and flip angle error; they have propagated to computed MWF maps. Our spiral maps also show heightened MWF in the basal ganglia compared to other reports. T2 shortening induced by iron deposition [44] [45], is known to relate linearly with R2 [44], [46], [47]. Our MWF results suggest specific shortening of extra-cellular T2 into the myelin range, heightening apparent MWF which should not be considered myelin-related. Although this paper is not concerned with sequence design, we recognize that its limitations can affect clinical applications. We intend to implement parallel imaging to further reduce scan time and achieve isotropic voxel size, and consider more sophisticated composite and adiabatic pulses which minimize inhomogeneity artifacts.

Certainly, bias and gradient issues result from the tradeoffs involved in speedy spiral acquisitions, but it is possible that the non-linear nature of our constrained model might be exacerbating it. Our approach requires explicitly fitting a fast and a slower relaxing component, hence in the presence of noise or artifacts could be more predisposed to finding a myelin signal even when one may not be present. We will consider other penalty functions to prevent this. Bayesian model order selection will be implemented to determine whether the data supports multiple peaks or single peaks. The proposed algorithm can be improved in other ways: replacing global, spatially invariant high-pass filter in the spatial term with locally adaptive smoothness penalties might better preserve tissue boundary. Plans to exploit edge-preserving spatial priors [48] are ongoing and will be submitted separately.

## Supporting Information

File S1 Contains Figure S1, rMSE of MWF computed using proposed method on brain simulation with different *μ_S_* at SNR  =  100 and 300 (*μ_N_*  = 0.013). The optimal value was found at 0.01 for both noise levels. Figure S2, Visual results for spatial constrained (*μ_N_* = 0.013) on brain simulation at SNR of 100 (top) and 300 (bottom), where *μ_S_* = 0.0001, 0.001, 0.01, 0.02, 0.1, respectively, from left to right. The ones within red boxes provide the best visual and numerical result, and show that the optimal value of this parameter is not overly sensitive to SNR. Figure S3, Convergence of the proposed algorithm. Left: MWF maps computed after 20, 30, 80 and 100 iterations on an *in vivo* example. Right: The numerical convergence of the cost function shows a classic pattern, whereby convergence is reached at 20 iterations, and further iterations do not appreciably reduce the cost. Although MWF maps begin looking reasonable in as few as 20 iterations, we chose 30 iterations to provide a margin of error. Figure S4, MWF maps computed from another *in vivo* MS patient scan. Top to bottom are MWF maps from conventional method, spatial constrained method and FLAIR images. Arrows in FLAIR images point to lesions. Figure S5, Left to right, single axial slice of a MS patient showing A] T2-weighted image, B] MWF map from conventional NNLS method, C] MWF map from spatially constrained Gaussian method, and D] MWF map reconstructed from sparse L1-regularized method.
(DOCX)
