# Supplementary material for: Multi-Compartment T2 Relaxometry Using a Spatially Constrained Multi-Gaussian Model
Source: PLoS One. 2014 Jun 4;9(6):e98391. doi: 10.1371/journal.pone.0098391 (PMC4045663; doi:10.1371/journal.pone.0098391)
Supplement: File S1 — Contains Figure S1, rMSE of MWF computed using proposed method on brain simulation with different μS at SNR = 100 and 300 (μN = 0.013). The optimal value was found at 0.01 for both noise levels. Figure S2, Visual results for spatial constrained (μN = 0.013) on brain simulation at SNR of 100 (top) and 300 (bottom), where μS = 0.0001, 0.001, 0.01, 0.02, 0.1, respectively, from left to right. The ones within red boxes provide the best visual and numerical result, and show that the optimal value of this parameter is not overly sensitive to SNR. Figure S3, Convergence of the proposed algorithm. Left: MWF maps computed after 20, 30, 80 and 100 iterations on an in vivo example. Right: The numerical convergence of the cost function shows a classic pattern, whereby convergence is reached at 20 iterations, and further iterations do not appreciably reduce the cost. Although MWF maps begin looking reasonable in as few as 20 iterations, we chose 30 iterations to provide a margin of error. Figure S4, MWF maps computed from another in vivo MS patient scan. Top to bottom are MWF maps from conventional method, spatial constrained method and FLAIR images. Arrows in FLAIR images point to lesions. Figure S5, Left to right, single axial slice of a MS patient showing A] T2-weighted image, B] MWF map from conventional NNLS method, C] MWF map from spatially constrained Gaussian method, and D] MWF map reconstructed from sparse L1-regularized method. (DOCX) [file pone.0098391.s001.docx]

SUPPLEMENTARY INFORMATION

Figure S1. rMSE of MWF computed using proposed method on brain simulation with different $\mu_{S}$ at SNR = 100 and 300 ($\mu_{N}$ = 0.013). The optimal value was found at 0.01 for both noise levels.


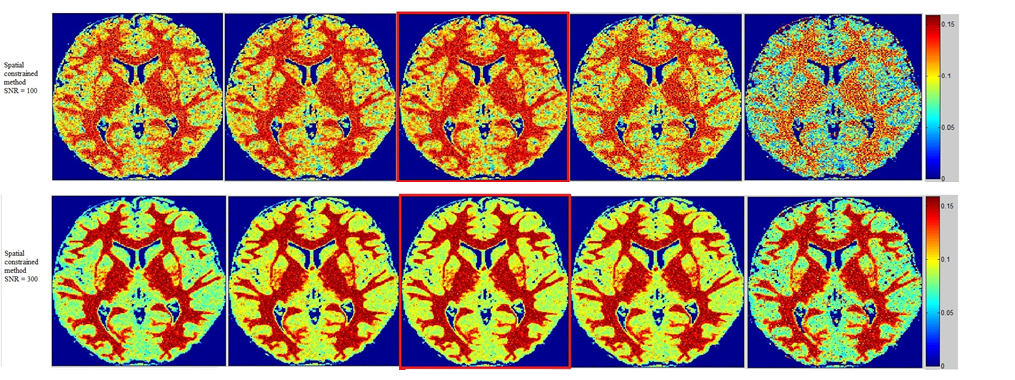


Figure S2. Visual results for spatial constrained ($\mu_{N}$ = 0.013) on brain simulation at SNR of 100 (top) and 300 (bottom), where $\mu_{S}$ = 0.0001, 0.001, 0.01, 0.02, 0.1, respectively, from left to right. The ones within red boxes provide the best visual and numerical result, and show that the optimal value of this parameter is not overly sensitive to SNR.


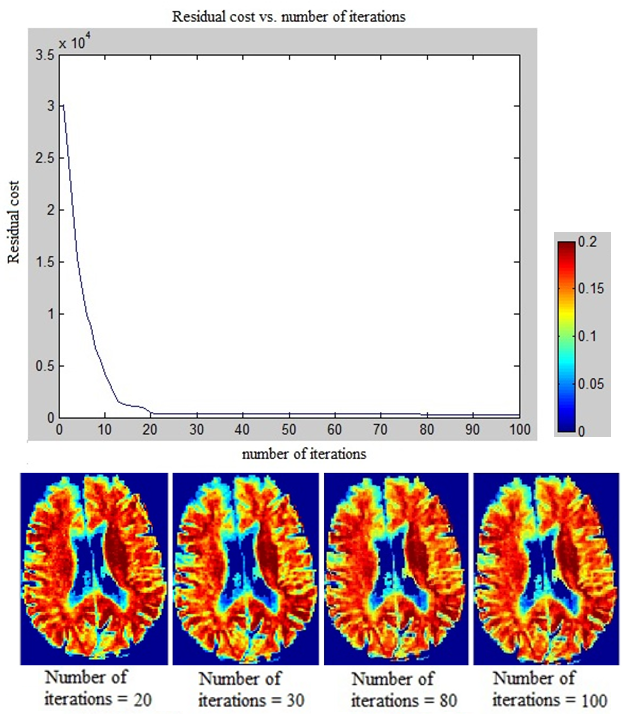


Figure S3: Convergence of the proposed algorithm. Left: MWF maps computed after 20, 30, 80 and 100 iterations on an *in vivo* example. Right: The numerical convergence of the cost function shows a classic pattern, whereby convergence is reached at 20 iterations, and further iterations do not appreciably reduce the cost. Although MWF maps begin looking reasonable in as few as 20 iterations, we chose 30 iterations to provide a margin of error.


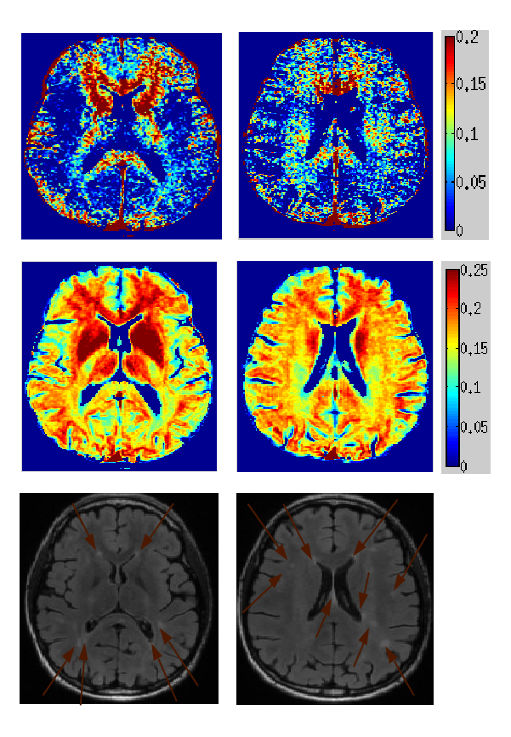


Figure S4. MWF maps computed from another *in vivo* MS patient scan. Top to bottom are MWF maps from conventional method, spatial constrained method and FLAIR images. Arrows in FLAIR images point to lesions.

## Supplementary Information S5

# Implementation and testing of Sparse Reconstruction using L1-norm penalty function

Since the T2 distribution of each voxel is given by a small number of tissue classes, it can be assumed to be sparse in the T2 range of interest, i.e. 5 ms to 300 ms. Therefore there is potential for using sparse reconstruction algorithms on this problem. In order to test this approach we implemented three sparse reconstruction methods: LI SPRIT, L1-magic and l1_ls with nonnegativity constraints, all three are cutting edge and widely used methods. These methods are able to handle sparsity-inducing L1-norm costs, and solve the optimization using semi-definite cone programming. We used l1_ls solver to solve large-scale l1-regularised least squares problem (LSPs) with non-negativity constraints. It solves LSPs using truncated newton interior-point method (IPM) as described by Kim et al [1]. L1_ls solver with non-negativity constraints solves an optimization problem by minimizing a function of the form

$\left| \left| Ax-y \right| \right|^{2}+ \lambda\sum_{i=1}^{n} x_{i}$ (1)

subject to $x_{i}\geq0 , i=1,\ldots,n$ for each voxel, where $A$ is data matrix of the form $A_{k,i}=exp\left( -{TE_{k}}/{T_{2}(i)} \right)$ at echo times TE_k_ (k = 1,..., K) and a set of i=1,…,N discrete sub-components which are hypothesized to exists as described in our manuscript. Vector $y$ is a collection of MR signals in a voxel acquired at each echo time TE_k,_ x is a vector of unknowns, and $\lambda$ is the regularization parameter. Our results, as applied to a single experimental image slice, are shown in Fig 1 below.


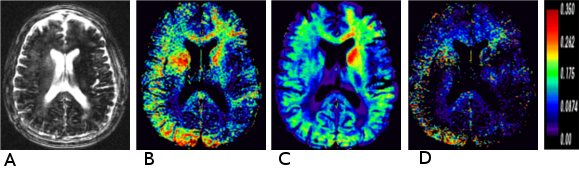


Figure S5: Left to right, single axial slice of a MS patient showing A] T2-weighted image, B] MWF map from conventional NNLS method, C] MWF map from spatially constrained Gaussian method, and D] MWF map reconstructed from sparse L1-regularized method.

As can be observed, the sparse result is no better than the conventional 50-point convex optimization using NNLS, and significantly worse than the 3-class model we have proposed. Please note that the sparse result shown here represents the best of a large set of empirical tweaking and parameter choices. The following parameter choices were attempted: $\lambda$, the L1 regularization parameter, was fixed, sequentially, at 1000, 100, 10, 1, 0.04, and 3-4 values in the range 1x10^-1^ to 1x10^-10^. The maximum number of IPM (Newton) iterations was varied as well; the following values were tried: 50, 100, 200, 300 and 400. The best results (from visual inspection and numerical range of MWF) was obtained at $\lambda$ = 0.01, number of iterations = 200. Single slice results are shown here, since the sparse method was too slow to be practically applied to whole brain volumes. An experimental run took 5 hours per slice, amounting to 140 hours for the whole brain.

The above results suggest that a direct application of sparse reconstruction methods is likely to be unsuccessful, since it is necessarily going to be done on a single voxel basis, just like the conventional NNLS case. This shows that imposing sparsity in T2 space is by itself not sufficient to overcome the ill-posedness of the problem. The proposed spatial approach is more successful because it favors both a model-based restriction to 3 classes, as well as additional spatial constraints.

**REFERENCES**

1. S.J. Kim, K. Koh, M. Lustig, S. Boyd, and D. Gorinevsky. A method for large-scale l1-regularized least squares. IEEE Journal on Selected Topics in Signal Processing, 1(4):606–617, 2007.
